# Supplementary material for: Association of Handgrip Strength with Diabetes Mellitus in Korean Adults According to Sex
Source: Diagnostics (Basel). 2022 Aug 2;12(8):1874. doi: 10.3390/diagnostics12081874 (PMC9406341; doi:10.3390/diagnostics12081874)
Supplement: Supplementary file 1 [file diagnostics-12-01874-s001.zip › diagnostics-1808553-supplementary.pdf]

Supplementary Table S1. Association between RGS (per 0.1kg) and the prevalence of DM in Koreans using logistic regression.

Men			Women (pre-menopause)			Women (post-menopause)		
	OR	*p*-value		OR	*p*-value		OR	*p*-value
Unadjusted	0.41 (0.37 – 0.45)	< 0.001	Unadjusted	0.21 (0.15 – 0.28)	< 0.001	Unadjusted	0.37 (0.31 – 0.43)	< 0.001
Model 1	0.59 (0.53 – 0.66)	< 0.001	Model 1	0.23 (0.17 – 0.32)	< 0.001	Model 1	0.51 (0.43 – 0.61)	< 0.001
Model 2	0.78 (0.70 – 0.88)	0.001	Model 2	0.66 (0.46 – 0.94)	0.022	Model 2	0.81 (0.67 – 0.99)	0.038
Model 3	0.80 (0.71 – 0.90)	0.007	Model 3	0.68 (0.47 – 0.98)	0.036	Model 3	0.84 (0.69 – 1.02)	0.080

Model 1: adjusted for age;

Model 2: adjusted for age, waist circumference, regular exercise, smoking status, and alcohol uptake

Model 3: adjusted for age, waist circumference, regular exercise, smoking status, alcohol uptake, total cholesterol, TG, AST, ALT and systolic blood pressure

Supplementary Table S2. Model diagnostics for logistic regression of Table 2.

	Pseudo R^2		
	Men	Women (pre-menopause)	Women (post-menopause)
Unadjusted	0.077	0.072	0.056
Model 1	0.174	0.134	0.077
Model 2	0.219	0.242	0.131
Model 3	0.257	0.287	0.220

Model 1: adjusted for age

Model 2: adjusted for age, waist circumference, regular exercise, smoking status, and alcohol uptake

Model 3: adjusted for age, waist circumference, regular exercise, smoking status, alcohol uptake, total cholesterol, TG, AST, ALT and systolic blood press

Supplementary Table S3. Model diagnostics for logistic regression of Table 3.

	Pseudo R^2		
	Men	Women (pre-menopause)	Women (post-menopause)
Unadjusted	0.071	0.068	0.053
Model 1	0.173	0.132	0.076
Model 2	0.220	0.248	0.131
Model 3	0.252	0.291	0.220

Model 1: adjusted for age

Model 2: adjusted for age, waist circumference, regular exercise, smoking status, and alcohol uptake

Model 3: adjusted for age, waist circumference, regular exercise, smoking status, alcohol uptake, total cholesterol, TG, AST, ALT and systolic blood press

Supplementary Table S4. Odds ratio and 95% confidence intervals for the prevalence of DM according to RGS quartile.

	Men				Women (pre-menopause)				Women (post-menopause)			
	Q_1	Q_2	Q_3	Q_4	Q_1	Q_2	Q_3	Q_4	Q_1	Q_2	Q_3	Q_4
	≤ 2.84	2.85–3.30	3.31–3.75	> 3.75	≤ 1.90	1.91–2.22	2.22–2.53	> 2.53	≤ 1.48	1.48–1.81	1.81–2.13	> 2.13
< 40 years												
Unadjusted	1.00	0.24 (0.13-0.44)	0.23 (0.11-0.52)	0.06 (0.02-0.15)	1.00	0.33 (0.16-0.66)	0.20 (0.08-0.48)	0.10 (0.03-0.31)	N/A	N/A	N/A	N/A
Model 1	1.00	0.22 (0.12-0.40)	0.20 (0.09-0.45)	0.05 (0.02-0.13)	1.00	0.29 (0.14-0.62)	0.17 (0.07-0.42)	0.08 (0.03-0.25)	N/A	N/A	N/A	N/A
Model 2	1.00	0.44 (0.22-0.86)	0.61 (0.27-1.36)	0.22 (0.07-0.67)	1.00	0.69 (0.29-1.63)	0.58 (0.21-1.59)	0.45 (0.13-1.55)	N/A	N/A	N/A	N/A
Model 3	1.00	0.38 (0.19-0.75)	0.61 (0.25-1.47)	0.21 (0.07-0.66)	1.00	0.84 (0.33-2.14)	0.70 (0.23-2.10)	0.54 (0.12-2.34)	N/A	N/A	N/A	N/A
40-59 years												
Unadjusted	1.00	0.67 (0.52-0.86)	0.58 (0.45-0.76)	0.28 (0.20-0.38)	1.00	0.50 (0.31-0.81)	0.48 (0.28-0.81)	0.06 (0.02-0.15)	1.00	0.71 (0.47-1.09)	0.57 (0.36-0.90)	0.39 (0.25-0.61)
Model 1	1.00	0.67 (0.52-0.86)	0.59 (0.46-0.77)	0.30 (0.22-0.42)	1.00	0.52 (0.32-0.83)	0.49 (0.29-0.85)	0.06 (0.02-0.17)	1.00	0.71 (0.47-1.08)	0.57 (0.36-0.90)	0.39 (0.25-0.61)
Model 2	1.00	0.82 (0.63-1.07)	0.85 (0.64-1.13)	0.49 (0.34-0.70)	1.00	0.81 (0.48-1.37)	1.23 (0.67-2.23)	0.19 (0.07-0.52)	1.00	0.82 (0.51-1.30)	0.87 (0.53-1.43)	0.87 (0.53-1.43)
Model 3	1.00	0.79 (0.60-1.04)	0.83 (0.62-1.12)	0.49 (0.33-0.71)	1.00	0.85 (0.50-1.44)	1.26 (0.68-2.33)	0.20 (0.07-0.55)	1.00	0.90 (0.55-1.46)	0.94 (0.56-1.56)	0.89 (0.53-1.49)
≥ 60 years												
Unadjusted	1.00	0.74 (0.61-0.90)	0.46 (0.36-0.59)	0.52 (0.36-0.75)	N/A	N/A	N/A	N/A	1.00	0.76 (0.62-0.92)	0.43 (0.34-0.54)	0.38 (0.30-0.50)
Model 1	1.00	0.70 (0.58-0.86)	0.43 (0.33-0.55)	0.47 (0.33-0.69)	N/A	N/A	N/A	N/A	1.00	0.84 (0.69-1.02)	0.51 (0.40-0.64)	0.49 (0.37-0.65)
Model 2	1.00	0.80 (0.65-0.98)	0.51 (0.38-0.68)	0.65 (0.43-0.97)	N/A	N/A	N/A	N/A	1.00	0.94 (0.76-1.17)	0.68 (0.53-0.87)	0.81 (0.60-1.10)
Model 3	1.00	0.83 (0.67-1.03)	0.55 (0.42-0.74)	0.74 (0.49-1.12)	N/A	N/A	N/A	N/A	1.00	0.92 (0.74-1.15)	0.69 (0.53-0.89)	0.84 (0.61-1.15)

Model 1: adjusted for age

Model 2: adjusted for age, waist circumference, regular exercise, smoking status, and alcohol uptake

Model 3: adjusted for age, waist circumference, regular exercise, smoking status, alcohol uptake, total cholesterol, TG, AST, ALT and systolic blood pressure

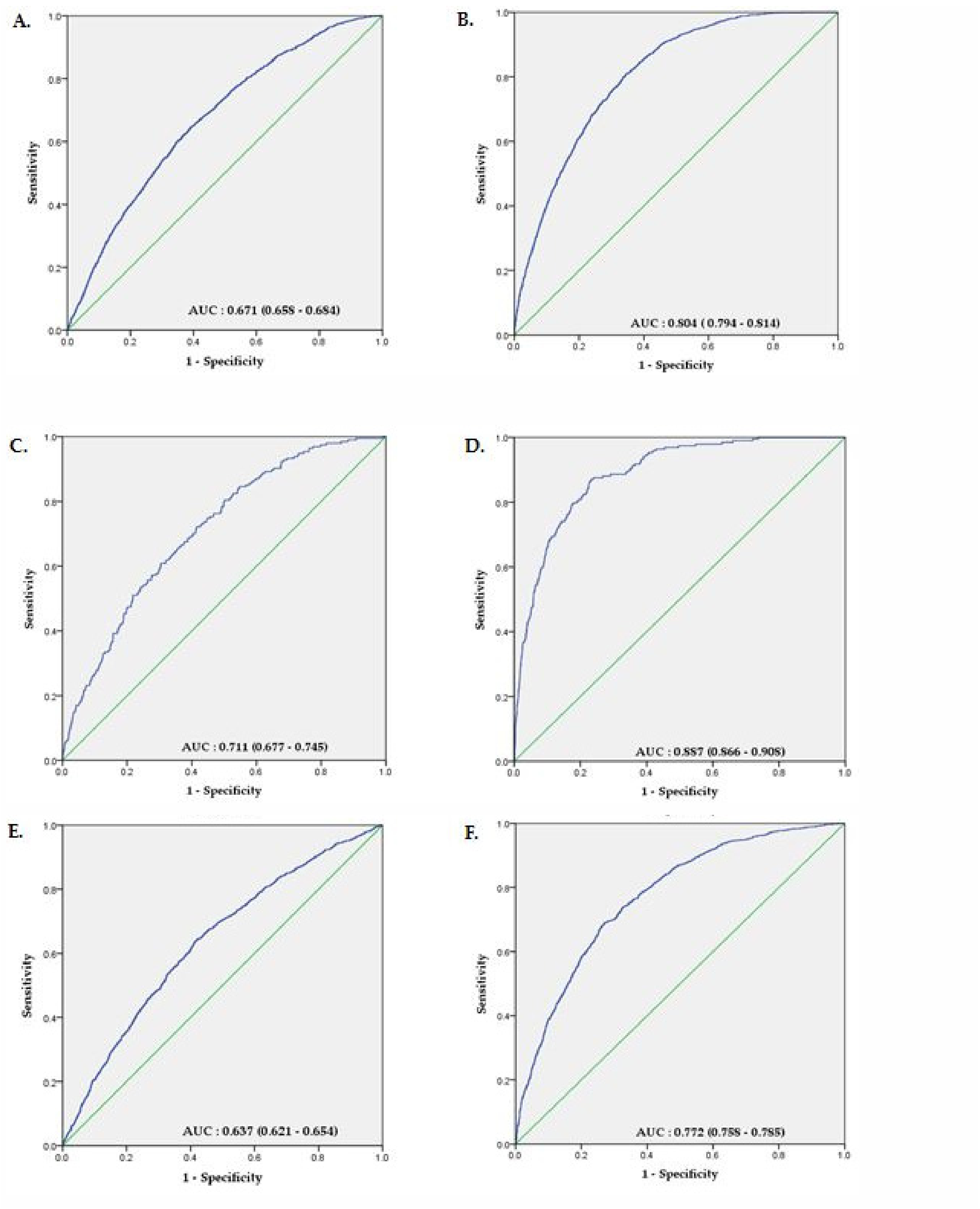

Supplementary Figure S1. ROC curve predicting prevalence of DM according to RGS (per 0.1kg) in men in unadjusted model (A), and model 3 (B), in premenopausal women in unadjusted model (C), and model 3 (D), in postmenopausal women in unadjusted model (E), and model 3 (F).
